# Nursing professionalism and associated factors in Ethiopia: a systematic review and meta-analysis

**DOI:** 10.1186/s12912-025-02713-w

**Published:** 2025-01-27

**Authors:** Moges Tadesse Abebe, Agerie Mengistie Zeleke, Yeshiwas Ayale Ferede, Yosef Aragaw Gonete, Worku Chekol Tassew

**Affiliations:** 1 Department of Pediatric and Child Health Nursing, College of Health Science, Debark University, Debark, Ethiopia; 2 Department of Clinical Midwifery, College of Health Science, Debark University, Debark, Ethiopia; 3 Department of Reproductive Health, Teda Health Science College, Gondar, Ethiopia; 4 Department of Midwifery, College of Health Science, Debark University, Debark, Ethiopia; 5 Department of Medical Nursing, Teda Health Science College, Gondar, Ethiopia

**Keywords:** Nursing, Professionalism, Associated factors, Ethiopia

## Abstract

**Background:**

A higher level of nursing professionalism improves autonomy among nurses, the quality of nursing care, and patient outcomes. However, inconsistent findings on the prevalence of nursing professionalism and associated factors have been reported among studies conducted in Ethiopia, and a meta-analysis of pooled results have not been performed. Therefore, the aim of this systematic review and meta-analysis was to determine the pooled prevalence of higher levels of nursing professionalism and factors associated with it.

**Methods:**

PubMed, Science Direct, HINARI, African Journals Online, Google Scholar, and university online institutional repositories in Ethiopia were accessed from 15/10/2024–30/10/2024. The items were assessed in accordance with the Preferred Reporting Items for Systematic Reviews and Meta-Analyses guidelines. The quality of the included studies was assessed via the Newcastle–Ottawa Scale. Cross-sectional studies were included without time period limits. Data extraction was conducted via Microsoft Excel and analyzed with STATA 17. The Galbraith plot, I^2^ statistic and meta-regression were used to determine heterogeneity. We used a random effects model in the presence of heterogeneity. Publication bias was assessed via funnel plots and Egger’s based regression. We also computed a sensitivity analysis and subgroup analysis by sample size and study period.

**Results:**

Twelve primary studies involving 3710 nurses were included in this systematic review and meta-analysis. The pooled prevalence of higher levels of nursing professionalism was 43%. Bachelor's degree and above educational status (POR: 1.80, CI: 1.38, 2.33), learning from government colleges (POR: 2.14, CI: 1.34, 3.42), better payment (POR: 1.85, CI: 1.16, 2.98), long years of work experience (POR: 2.15, CI: 1.73, 2.68), positive self-image (POR: 3.85, CI: 2.17, 6.84), job satisfaction (POR: 2.42, CI: 1.49, 3.95) and training opportunities (POR: 2.88, CI: 1.14, 7.32) were factors that determined higher levels of nursing professionalism in Ethiopia.

**Conclusion:**

The pooled prevalence of higher levels of nursing professionalism in Ethiopia was low. Educational status, and attending college, payment, work experience, self-image, job satisfaction, and training were factors that determined the level of professionalism. These factors can be modified to increase the level of nursing professionalism in Ethiopia.

**Supplementary Information:**

The online version contains supplementary material available at 10.1186/s12912-025-02713-w.

## Introduction

A professional is an individual that upholds ethical standards and is regarded by the public as having unique knowledge and abilities in a body of knowledge that is widely acknowledged to have been obtained through high-level research, education, and training, and who is willing to use this knowledge and exercise these abilities for the benefit of others [1, 2]. A knowledge base, a well-defined monopoly over a field of work, and autonomy or control over work are the components of a professional [3].

Professionalism is a multidimensional concept that encompasses individual, interpersonal, and social interactions. It is defined as a skill, judgment, and polite behavior that is expected from a person who is trained to perform a job [4, 5]. It also explains the extent to which an individual identifies with a profession and adheres to its standards. Professionalism also defines the code of conduct, professional relationships, competence, and communication skills [6, 7]. The basic idea of professionalism, which has been brought forward by various societies, is the notion of the duties, qualities, interactions, attitudes, and role behaviors that professionals must have with both individual clients and society at large, or it is the behavior or attributes that characterize a professional or a career [8]. The integrated beliefs, concepts, and values for nursing and nurses as a profession are known as professionalism in nursing [9, 10]. Nursing professionalism encompasses knowledge, the spirit of inquiry, autonomy, accountability, innovation, advocacy and vision, collaboration and collegiality, and ethics [6].

Research has indicated that nursing professionalism improves the professional knowledge of nurses and their skill and organizational productivity. Higher levels of nursing professionalism can improve autonomy among nurses and empower them, advance their recognition, alleviate organizational citizenship behaviors, enhance the standard of nursing care and improve nursing care quality [11, 12].

Nursing professionalism is considered a vital component of quality nursing care improvement strategies globally, and studies have attempted to address its level and determine its factors. In this context, the studies conducted in Japan and Turk revealed a lower level of nursing professionalism, and the factors that affected nursing professionalism were level of education, years of experience, and position [13–15]. However, Iranian nurses have a moderate level of nursing professionalism [16, 17]. In another study conducted among nurses, the factors that determined nursing professionalism were professional organization, position, employment status, work setting, years of experience, and degree of attainment. Among Korean and Chinese nurses, nursing professionalism is the major determinant that affects job satisfaction [18, 19]. Work experience was found to influence nursing professionalism in another study conducted in Japan [20]. The nursing work environment was also found to be a determinant of nursing professionalism [21]. Being a member of a professional association and having long years of experience were factors that determined nursing professionalism in another study [22].

Among the primary studies conducted in Ethiopia, the level of nursing professionalism has been inconsistently reported; most of the studies reported it to be low, and few studies reported a moderate to high level of nursing professionalism. The prevalence of higher levels of nursing professionalism according to primary studies conducted in Ethiopia widely varies from 12.86% to 58.68%. Various variables were also analyzed to identify the factors that determine nursing professionalism in Ethiopia. Among them, being a female nurse, being older than 40 years, having better payments, having a long year of experience, nursing association membership, positive self-image, higher educational status, job satisfaction, good organizational culture, self-image and having training opportunities are the most common factors associated with nursing professionalism in Ethiopia [23–34].

To the best of our knowledge, no meta-analysis or systematic review has been done on the prevalence of higher levels of nursing professionalism and associated factors in Ethiopia and throughout Africa. In order to ascertain the combined prevalence of higher levels of nursing professionalism and related factors in Ethiopia, this systematic review and meta-analysis was conducted. To find the gaps in the level of nursing professionalism, pooled data demonstrating the prevalence and variables related to nursing professionalism are required. Policymakers and nursing leaders can utilize the information in this report to design and oversee nursing programs in Ethiopia. By implementing evidence-based nursing care in Ethiopia, the findings may also contribute to better patient outcomes and nursing care delivery. Therefore, the aim of this systematic review and meta-analysis was to determine the pooled prevalence of higher levels of nursing professionalism and associated factors among nurses in Ethiopia.

## Methods

### Study design

Systematic review and meta-analysis.

### Study setting

The included primary studies were conducted among nurses working in health institutions of Ethiopia. The Ethiopian nursing association, which is a legally recognized professional nursing association, represents more than 68,961 nurses working in the country [35]. The number of nurses and midwives was 0.8 for 1000 people, based on the World Bank report of December 2024.

### Reporting and searching

This systematic review was reported according to the Preferred Reporting Items for Systematic Reviews and Meta-Analyses (PRISMA, 2020) guidelines [36] **(**S1 File**)**. Before database searching, we have assessed the prospective register of systematic reviews (PROSPERO) to check for duplicated work, and there was no registered review with our title. Then, the PubMed, Science Direct, HINARI, African Journals Online (AJOL), online universities repository in Ethiopia, and Google Scholar databases were accessed from 15/10/2024–30/10/2024. The references of the articles retrieved were also searched to avoid missing any article from different sources. Boolean operators AND/OR combine the keywords nursing, professionalism, associated factors, and Ethiopia. Synonymous and medical subject heading (MeSH) terms were also used to search the databases (S2 File).

The research questions were constructed using condition, outcome, context, population (CoCOPop) strategies.

### Condition

The prevalence of higher level of nursing professionalism and associated factors.

### Context

Ethiopia.

### Outcomes

The primary outcome was the pooled prevalence of a higher level of nursing professionalism, and the secondary outcomes were factors associated with a higher level of nursing professionalism. A higher level of nursing professionalism was determined on the basis of those nurses who scored above the mean score of the professionalism questions for binary outcome studies, and those nurses who scored high among the tertile ranked studies categorized as low, medium and high.

### Population

Nurses.

### Inclusion criteria

We included cross-sectional studies conducted on the prevalence of nursing professionalism or associated factors in Ethiopia. The articles included were either published or unpublished without time bounds, but were restricted to the English language.

### Exclusion criteria

We excluded studies conducted with nursing students about professionalism because nursing students are not licensed in the nursing profession. We also excluded articles that were not fully accessible and published in language other than English. Additionally, studies that lacked our outcome measures on the prevalence of nursing professionalism and associated factors in Ethiopia were excluded.

### Study selection and critical appraisal

After database searching, we exported the data to the EndNote 7 citation manager and removed duplicates. Titles and abstracts were independently screened by two reviewers (MT and WC). Discrepancies among the two reviewers were subsequently resolved by consulting a third reviewer (AM) and a thorough revision and discussion among the team members to achieve a consensus. We used the Newcastle–Ottawa scale (NOS) quality assessment checklist for cross-sectional studies to assess the quality of the included articles [37]. We included articles scoring 7 or more out of the 10 assessment criteria. Reviewers MT and YA critically appraised each article independently, and any discrepancy noted was resolved with another third reviewer (WC) (S3 File).

### Data extraction

We used a Microsoft Excel 2010 spreadsheet to extract the data (S4 files). The first author’s last name, study year, sample size, study region, odds ratio (OR) with 95% confidence interval (CI), frequency of highest level of nursing professionalism, response rate, and NOS scale were extracted (Table 1). Two reviewers (MT and WC) extracted the data independently, crosschecked for any discrepancy, and then, resolved the data with discussion [38].

**Table 1:** Characteristics of the included studies on higher levels of nursing professionalism in Ethiopia

Author (year of study)	Region	Frequency of higher professionalism	Sample size	Prevalence of higher professionalism	Response rate %	NOS Scale
Rekisso et al. 2022 [29]	AA	161	348	46.26	97.5	9
Solomon et al. 2015 [30]	Oromia	88	303	29.04	91.2	7
Boe et al. 2024 [24]	Sidama	110	405	27.16	98	9
Bekalu et al. 2022 [25]	Amhara	240	350	68.57	98	9
Getu et al. 2015 [28]	Amhara	54	103	52.43	98.1	7
Fantahun et al. 2014 [26]	Tigray	27	210	12.86	94.6	7
Tura et al. 2022 [33]	Oromia	130	238	54.62	97.9	8
Abate et al. 2021 [23]	Amhara	101	407	24.82	99.3	9
Mengesha et al. 2022 [31]	Central	104	360	28.89	95.7	8
Fentaw et al. 2022 [27]	Amhara	181	350	51.71	98	9
Wondwossen et al. 2011 [34]	AA	145	256	56.64	96	8
Tola et al. 2018 [32]	Oromia	223	380	58.68	90.5	8

*AA* Addis Ababa

### Data analysis

We used STATA 17 statistical software for data analysis. The heterogeneity of the included articles was assessed via the I-squared (I^2^) statistic. We used the fixed effects model when the I^2^ values were less than 50%, and the random effects model was used when the I^2^ values were greater than 50%. Additionally, meta-regression and the Galbraith plot were also executed. Furthermore, Egger’s-based test and funnel plots regression were assessed to determine publication bias among the included studies. We performed a sensitivity analysis to examine the effect of a single study on the overall pooled result. We also conducted subgroup analysis by sample size and study period to identify their effects on heterogeneity [39].

## Results

### Selection results

**Fig. 1:**
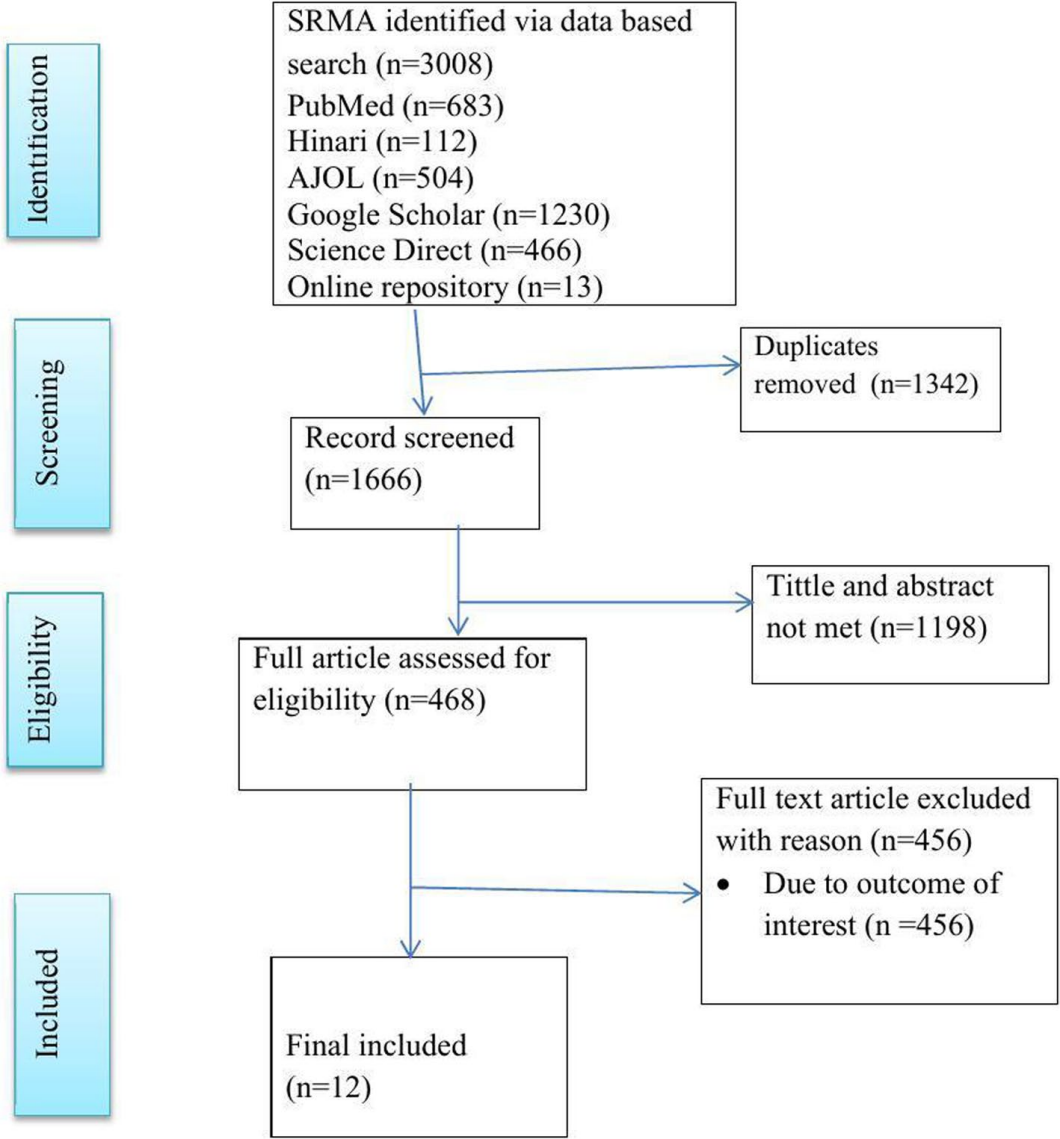
PRISMA flow diagram of study selection for higher levels of nursing professionalism in Ethiopia

Overall, 3008 articles were retrieved from online databases. After being exported to the EndNote 7 citation manager, 1342 duplicates were removed, and 1198 were excluded after title and abstract screening. A total of 455 full articles were assessed for eligibility; 456 were excluded because of outcomes of interest. Finally, 12 articles were included in this systematic review and meta-analysis [23–34] (Fig. 1).

### Characteristics of the included studies

Twelve cross-sectional studies with a total of 3710 nurses were involved in this review. Among them, four were unpublished studies [27, 31, 32, 34]. Four studies were from the Amhara region [23, 25, 27, 28], two studies were from Addis Ababa [29, 34], three studies were from the Oromia region [30, 32, 33], one study was from the Tigray region [26], one study was from the Sidama region [24], and one study was from the central Ethiopian region [31]. The smallest and largest sample sizes were 103 [28] and 407 [23], respectively (Table 1).

### Pooled prevalence of higher levels of nursing professionalism

**Fig. 2:**
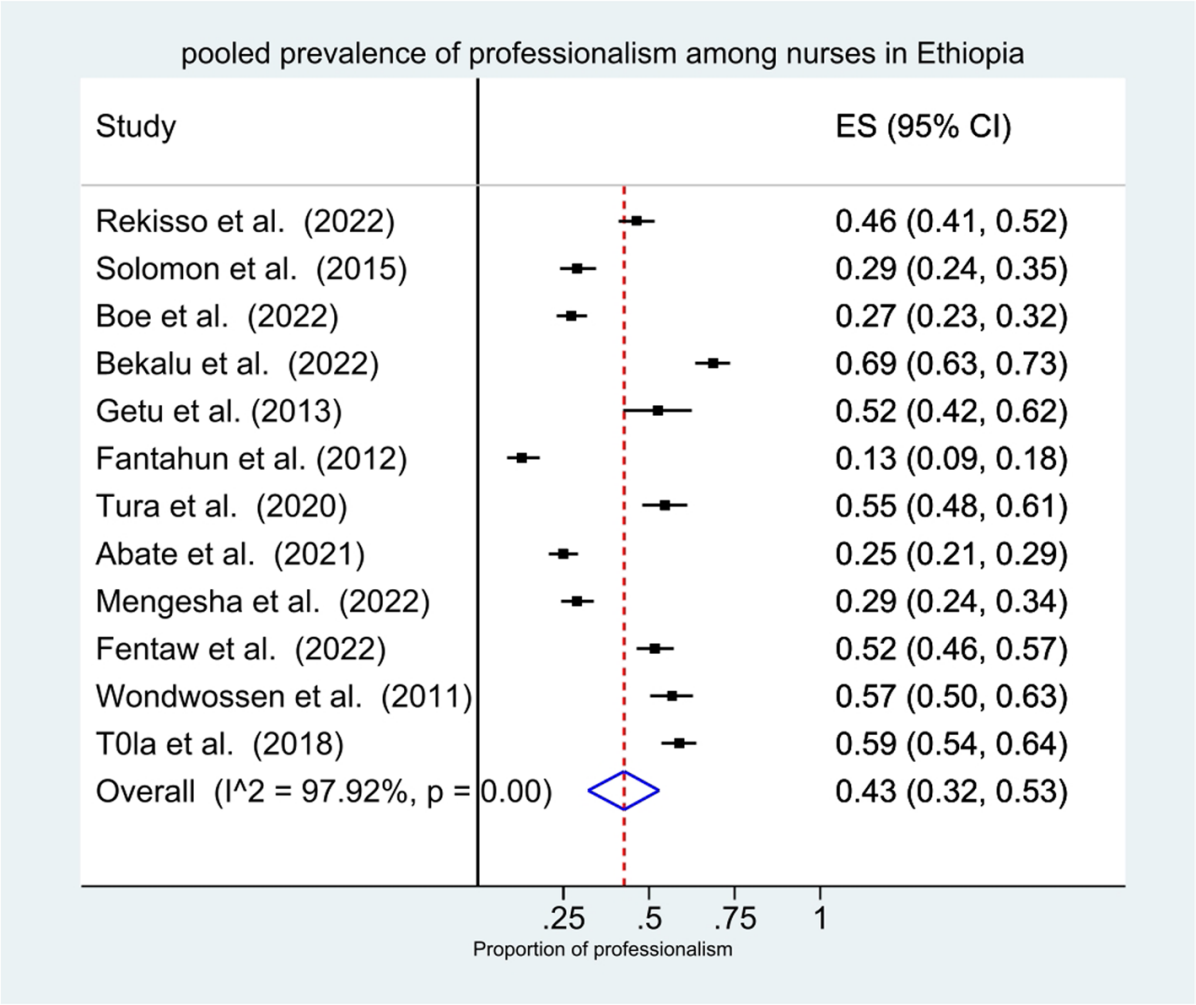
Forest plot showing the pooled prevalence of higher levels of nursing professionalism in Ethiopia

The pooled prevalence of higher levels of nursing professionalism according to the random effects model was 43% (CI: 32%, 53%), I^2^=97.92%, *p*<0.001 (Fig. 2).

### Publication bias and heterogeneity test

**Fig. 3:**
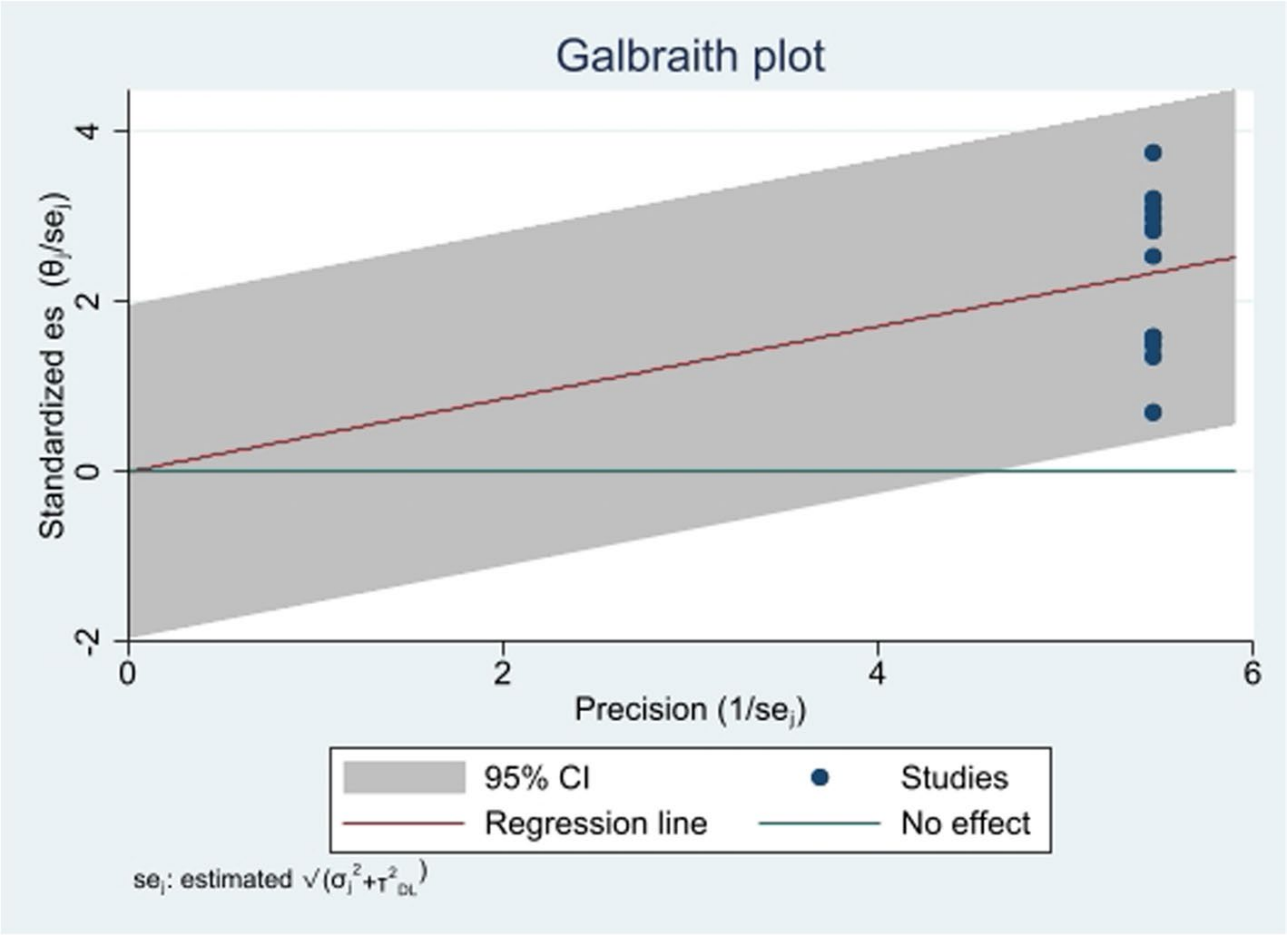
Galbraith plot showing heterogeneity of higher levels of nursing professionalism in Ethiopia

The funnel plot results revealed an asymmetric distribution, but Egger’s based regression analysis revealed a *p*-value of 0.245, which indicated lack of publication bias among studies. Furthermore, the Galbraith plot indicated that all studies had CIs of 95%, which confirmed the absence of any outliers (Fig. 3). We also computed a meta-regression analysis considering sample size and study year as covariates which could identify heterogeneity sources. However, there was no significant influence of heterogeneity among these covariates (Table 2).

**Table 2:** Meta-regression analysis of heterogeneity among studies of higher levels of nursing professionalism in Ethiopia

Source of heterogeneity	Coefficients	Standard errors	*p*-values
Study year	-0.0007272	0.0009484	0.463
Sample size	0.0149987	0.0197605	0.467

### Subgroup analysis

We also conducted subgroup analysis on the basis of the study period before and after 2019, by a sample size of less than 350 and greater than or equal to 350 and by study region. Hence, the pooled prevalence of higher levels of nursing professionalism did not significantly differ by study period and sample size. By region, it was greater than the pooled result in Addis Ababa, Amhara and Oromia regions, but it was lower than the pooled result in Tigray, Sidama and Central Ethiopian regions (Table 3).

**Table 3:** Pooled subgroup analysis results by study period, sample size and region for higher levels of nursing professionalism in Ethiopia

Subgroups	Categories	Pooled results
By study period	Before 2019	42
	After 2019	43
By sample size	Less than 350	42
	Greater than or equal to 350	44
By region	Addis Ababa	51
	Oromia	47
	Sidama	27
	Amhara	49
	Tigray	13
	Central	29

### Sensitivity analysis

**Fig. 4:**
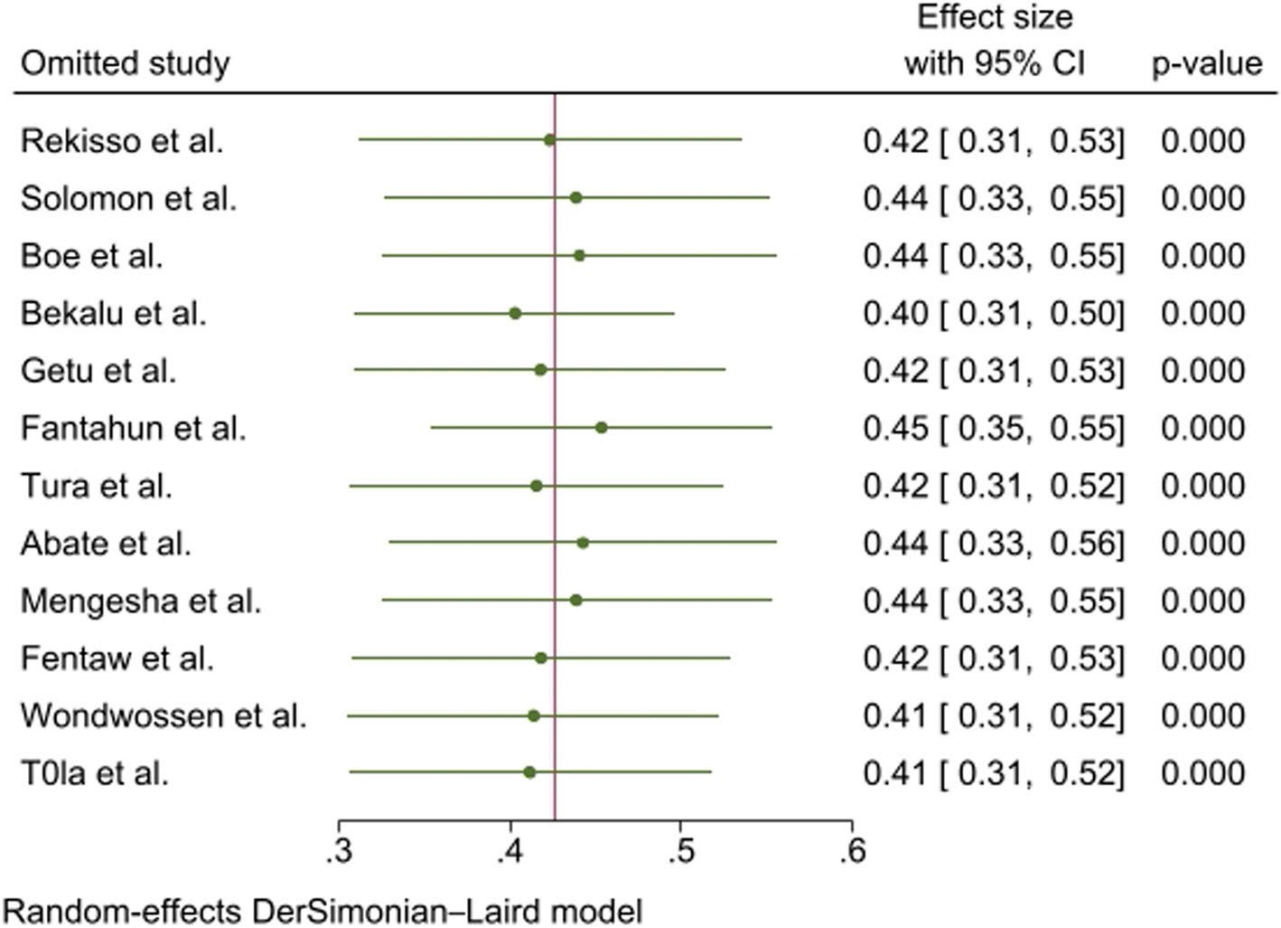
Sensitivity analysis results of higher levels of nursing professionalism in Ethiopia

We also conducted a sensitivity analysis by removing one study at a time to examine the influence of each study on the pooled result, but there was no single study influence on the overall effect size (Fig. 4).

### Factors associated with a higher level of nursing professionalism

The factors associated with higher levels of nursing professionalism were education, payments, work experience, self-image, satisfaction and training (Table 4).

**Table 4:** Factors associated with higher level of nursing professionalism in Ethiopia

Determinants (references)	Number of studies	Sample size	OR (95% CI)	*P*-Value	I^2^ (%)	Heterogeneity test (*p*-value)	Egger’s test (*p*-value)	Model used
**BSc and above** [23, 29, 32, 34]	**8**	**2487**	**1.80 (1.38, 2.33)**	**<0.001**	**0**	**0.499**	**0.22**	**Fixed effect**
**Government college** [25, 31]	**2**	**454**	**2.14 (1.34, 3.42)**	**<0.001**	**0**	**0.343**	**-**	**Fixed effect**
**Long year of Experience** [23, 29, 33, 34]	**4**	**1373**	**2.15 (1.73, 2.68)**	**<0.001**	**25**	**0.259**	**0.084**	**Fixed effect**
**Better payment** [23, 28, 29, 32–34]	**6**	**1732**	**1.85 (1.16, 2.98)**	**0.01**	**13.2**	**0.33**	**0.67**	**Fixed effect**
**Positive self-image** [25, 27]	**2**	**421**	**3.85 (2.17, 6.84)**	**<0.001**	**20.1**	**0.263**	**-**	**Fixed effect**
**Satisfied** [25, 27, 32]	**3**	**644**	**2.42 (1.49, 3.95)**	**<0.001**	**64.9**	**0.058**	**0.11**	**Random effect**
**Received training** [23, 31]	**2**	**327**	**2.88 (1.14, 7.32)**	**<0.001**	**84.2**	**0.017**	**-**	**Random effect**

Nurses with BSc and above educational status had a higher level of nursing professionalism than diploma nurses did (POR: 1.80, CI: 1.38, 2.33), I^2^=0%, *p*=0.499. Eight primary studies with 2487 nurses were included in this category of factor analysis.

Additionally, nurses who learned from government colleges had a higher level of nursing professionalism than those who learned from private college did (POR: 2.14, CI: 1.34, 3.42), I^2^=0%, *p*=0.343. Two primary studies with 454 nurses were included in this category of analysis.

Nurses with long year of work experience had a higher level of nursing professionalism than did nurses with short periods of work experience (POR: 2.15, CI: 1.73, 2.68), I^2^=25%, *p*=0.259. Four primary studies with 1373 nurses were included in this category of analysis.

Nurses who received better payments had a higher level of professionalism than those who received smaller payments did (POR: 1.85, CI: 1.16, 2.98), I^2^=13.2%, *p*=0.33. Six primary studies with 1732 nurses were included in this factor analysis.

Nurses who had positive self-images also had higher levels of professionalism than those who had negative self-images did (POR: 3.85, CI: 2.17, 6.84), I^2^=20.1%, *p*=0.263. Two primary studies with 421 nurses were included in this category of analysis.

Satisfied nurses had a higher level of nursing professionalism than unsatisfied nurses did (POR: 2.42, CI: 1.49, 3.95), I^2^=64.9%, *p*=0.058. Three primary studies with 644 nurses were included in this category of analysis.

Finally, nurses who received training had a higher level of nursing professionalism than those who did not receive training did (POR: 2.88, CI: 1.14, 7.32), I^2^=82.4%, *p*=0.017. Two primary studies with 327 nurses were included in this category of analysis.

## Discussion

The aim of this meta-analysis was to determine the pooled prevalence of higher levels nursing professionalism and its associated factors in Ethiopia. The pooled prevalence of higher levels of nursing professionalism in Ethiopia was 43%. This meta-analysis revealed that a small number of nurses in Ethiopia had relatively high level of nursing professionalism because fewer than 50% of the nurses had good nursing professionalism. A lower level of nursing professionalism affects nursing care provision, which could result in poor patient outcomes.

Although the pooled prevalence of higher levels of nursing professionalism did not significantly differ by study period and sample size, it was greater in the Addis Ababa, Amhara, and Oromia regions, but it was lower in the Tigray, Sidama, and Central Ethiopian regions. These regional variations could possibly be due to differences in the factors that affect the level of nursing professionalism in different parts of Ethiopia. For example, more experienced nurses are moving to better-developed areas such as Addis Ababa to work and live there. Benefit package variation among different regions of the country may also have contributed to the regional variation of higher levels of nursing professionalism. The variation in the number of included articles could be the other reason for the discrepancies in the pooled prevalence of higher levels of nursing professionalism.

The various factors that lead to a higher level of nursing professionalism are discussed below. Improving the modifiable factors to increase the level of nursing professionalism is vital to improve nursing care in Ethiopia.

It is possible to enhance the level of nursing professionalism simply by providing education and training opportunities for nurses. Nurses with a BSc and above had a 1.8 times higher level of professionalism than diploma nurses did. Furthermore, nurses who received training opportunities were 2.88 times more likely to have higher levels of nursing professionalism. These findings were supported by systematic reviews conducted in Iran and America [40, 41]. These findings could be due to education and training providing a sense of understanding and ownership of professional responsibility for nurses. The more the nurse is knowledgeable about his profession, the more s/he can have a higher level of professionalism [22].

A higher level of nursing professionalism was also affected by the payment that nurses received. The nurses with better payments had 1.85 times a greater level of professionalism. Compared with those in other countries, the payments of Ethiopian nurses are lower, which could be the main reason for the poor healthcare delivery system in Ethiopia. The nurses with highest work load burden should have appropriate compensatory payments that improve the life of each nurse so that nursing care can be improved [42].

The level of nursing professionalism was also determined by nurses’ job satisfaction level. Satisfied nurses were 2.42 times more likely to have a higher level of nursing professionalism than unsatisfied nurses were. The level of professionalism is directly related to job satisfaction; thus, enhancing the level of nursing professionalism by resolving factors that affect nurses’ job satisfaction is possible [18, 19, 43, 44].

Nurses who had worked for a long period of year were also 2.15 times more likely to have higher levels of nursing professionalism than those who had worked for a short period of year. The possible justification could be that work experience increases, the opportunity for better payment and education, as well as training, might have increased, which has led to a higher level of professionalism among nurses. Owing to the high turnover rates among nurses in Ethiopia, it has been difficult to retain employees with long years of experience in a particular organization, which has led to the hiring of new staff with less nursing professionalism [45].

The positive self-image of nurses led to a 3.85 times higher level of nursing professionalism than did the negative self-image. Self-image builds confidence among nurses, where it can have a positive effect on the level of nursing professionalism [46].

The institution where nurses learn their education determines the level of professionalism. Those nurses who learned their education from government colleges had a 2.14 times higher level of nursing professionalism than those who learned from private colleges. This might be explained by the business-oriented nature of private colleges, where there is no adequate infrastructure, which may reduce the quality of education. This could have a negative effect on the knowledge and skills of nurses. To achieve a higher level of nursing professionalism and to adhere to nursing care standards as well as to provide high quality nursing care, a strong, powerful basis in theory, practice, and professional education in the nursing discipline is needed because they are a basis for establishing professionalism among nurses [47].

### Limitations of the study

The included studies did not represent all regions of the country, which may result in a lack of country representativeness. Although we used the random effects model, there was high heterogeneity across the studies included. Only English-language literature was included, which could be another limitation of this review. Furthermore, none of the included articles demonstrated a cause-effect association because they were all cross-sectional in design.

### Implications of the study

This meta-analysis can enhance nursing care provisions, improve patient outcomes, inform policies, and foster evidence-based nursing practices through enhancing nursing professionalism in Ethiopia. In this review, health institutions can develop and implement strategies to improve the level of nursing professionalism. It can also contribute to the global body of knowledge and practices related to the level of nursing professionalism and associated factors. This review highlights the gaps that need to be filled by nurse managers to enhance their employees’ sense of professionalism by resolving the factors that reduce the level of nursing professionalism. It can also indicate the area of research, especially the need to explore different factors in a qualitative study design. This meta-analysis also highlights the need to evaluate and update the nursing curriculum and books to include nursing professionalism concepts. Hence, it can contribute to educational reform by indicating the need for including broader concepts of nursing professionalism in nursing education and curriculum. Additionally, policymakers may also benefit from the findings in designing different nursing programs around the country.

### Conclusions and recommendations

The pooled prevalence of a higher level of nursing professionalism was low, since less than 50% of nurses had a higher level of nursing professionalism. The educational status and the college they attend, payment, work experience, self-image, satisfaction, and training are factors that determine the level of professionalism. These factors can be modified to increase the level of nursing professionalism in Ethiopia.

Nursing managers should facilitate better payment and satisfaction mechanisms and retain more experienced nurses in their organization for a higher level of nursing professionalism. They should also facilitate educational and training opportunities for nurses to achieve a higher level of professionalism. The ministry of health should monitor private colleges regularly for their focus on the concept of nursing professionalism for the students. The Ethiopian nursing association should work to enhance the level of nursing professionalism by addressing the factors that affect it. Researchers need to focus on the qualitative inquiry of nursing professionalism to investigate additional factors that affect it.

## Supplementary Information

Supplementary file 1. PRISMA 2020 checklist.

Supplementary file 2. Search strategy.

Supplementary file 3. NOS appraisal checklist.

Supplementary file 4. Data extracted

## Data Availability

Data is provided within the manuscript or supplementary information files.

## Abbreviations

BSc: Bachelor’s of Science
CI: Confidence Interval
NOS: Newcastle–Ottawa Scale
POR: Pooled Odds Ratio
PRISMA: Preferred Reporting Items for Systematic Review and Meta-analysis
