# Supplementary material for: Nursing professionalism and associated factors in Ethiopia: a systematic review and meta-analysis
Source: BMC Nurs. 2025 Jan 27;24:95. doi: 10.1186/s12912-025-02713-w (PMC11770932; doi:10.1186/s12912-025-02713-w)
Supplement: Supplementary file 2 — Search strategy. [file 12912_2025_2713_MOESM2_ESM.docx]

Newcastle-Ottawa Scale for included studies included in nursing professionalism in Ethiopia

|  | **Study selection** | | | | **Compatibility** | | **Outcome** | | **Total score** |
| --- | --- | --- | --- | --- | --- | --- | --- | --- | --- |
| **Study** | **Representativeness of sample size (**)** | **Sample size (*)** | **Non-respondents (*)** | **Ascertainment**  **(**)** | **The study Controls most important factors (*)** | **The study Controls any additional factor (*)** | **Ascertainment (*)** | **Statistical test (*)** |  |
| \| Rekisso et al. \| \| --- \| | 2 | 1 | 1 | 1 | 1 | 1 | 1 | 1 | 9 |
| \| Solomon et al. \| \| --- \| | 2 | 1 | 0 | 1 | 1 | 1 | 0 | 1 | 7 |
| \| Boe et al. \| \| --- \| | 1 | 1 | 1 | 2 | 1 | 1 | 1 | 1 | 9 |
| \| Bekalu et al. \| \| --- \| | 2 | 1 | 1 | 1 | 1 | 1 | 1 | 1 | 9 |
| \| Getu et al. \| \| --- \| | 1 | 0 | 1 | 1 | 1 | 1 | 1 | 1 | 7 |
| Fantahun et al. | 0 | 1 | 1 | 1 | 1 | 1 | 1 | 1 | 7 |
| Tura et al. | 1 | 1 | 1 | 1 | 1 | 1 | 1 | 1 | 8 |
| Abate et al. | 2 | 1 | 1 | 1 | 1 | 1 | 1 | 1 | 10 |
| Mengesha et al. | 1 | 1 | 1 | 1 | 1 | 1 | 1 | 1 | 8 |
| Fentaw et al. | 2 | 1 | 1 | 2 | 1 | 0 | 1 | 1 | 9 |
| Wondwossen et al. | 1 | 1 | 1 | 2 | 1 | 1 | 1 | 1 | 8 |
| T0la et al. | 2 | 1 | 0 | 1 | 1 | 1 | 1 | 1 | 8 |
