# Supplementary material for: Nursing professionalism and associated factors in Ethiopia: a systematic review and meta-analysis
Source: BMC Nurs. 2025 Jan 27;24:95. doi: 10.1186/s12912-025-02713-w (PMC11770932; doi:10.1186/s12912-025-02713-w)
Supplement: Supplementary file 3 — NOS appraisal checklist. [file 12912_2025_2713_MOESM3_ESM.docx]

S2 file: Search Strategies and number of articles retrieved among included databases for nursing professionalism in Ethiopia.

| Data base | Search query | Search results |
| --- | --- | --- |
| PubMed | ((((Nursing OR nurse OR nurses’ OR nurse’s OR nurses) [MeSH Terms]) AND ((professionalism OR profession) [MeSH Terms])) AND ((associated factors OR determinants OR risk factors))) AND (Ethiopia) | 683 |
| Science direct | (Nursing) AND (associated factors) AND (professionalism OR profession) AND Ethiopia | 466 |
| HINARI | Nursing OR nurse OR nurses’ OR nurse’s OR nurses) AND (associated factors OR determinants OR risk factors) AND (professionalism OR profession) AND Ethiopia | 112 |
| AJOL | (Nursing OR nurse OR nurses’ OR nurse’s OR nurses) AND (associated factors OR determinants OR risk factors) AND (professionalism OR profession) AND Ethiopia | 504 |
| Google scholar | (Nursing OR nurse OR nurses’ OR nurse’s OR nurses) AND (associated factors OR determinants OR risk factors) AND (professionalism OR profession) AND Ethiopia | 1230 |
